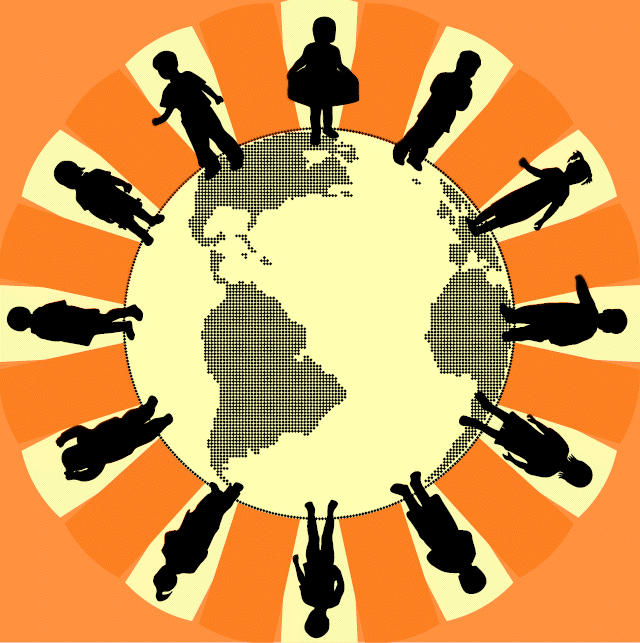# EHPnet: Global Initiative on Children’s Environmental Health Indicators

**Published:** 2006-10

**Authors:** Erin E. Dooley

More than 22,000 people came together in the late summer of 2002 in Johannesburg, South Africa, for the UN World Summit on Sustainable Development. According to *Using Indicators to Measure Progress on Children’s Environmental Health: A Call to Action*, a report presented at the summit, 2 million children under the age of 15 perish due to acute respiratory infections each year, diarrheal diseases cause the deaths of just under another 2 million, and more than 1 million children succumb to malaria. The majority of these disease cases are attributable to poor environmental conditions. A number of significant commitments and initiatives were agreed upon during the summit in a bid to combat these figures. One of these was the Global Initiative on Children’s Environmental Health Indicators, led by the WHO and described on its website at **http://www.who.int/ceh/indicators/globinit/en/index.html**.

The initiative homepage outlines the effort’s three main objectives: to formulate and advocate the use of children’s environmental health indicators, to improve ways to assess children’s environmental health and monitor how well interventions are working, and to work with policy makers to improve the environments where children live. This page also provides background information on how the initiative works.

The initiative will launch a series of regional pilot projects to develop children’s environmental health indicators, collect data for these indicators, and use the findings to better inform policy making at all levels of government. The leaders of each regional project choose for themselves how to approach these tasks. The initiative encourages the use of low-cost approaches that take advantage of existing data and indicators, and that work toward “a more harmonized and complete assessment of the state of children’s environmental health in the longer term.”

A pdf version of *Using Indicators to Measure Progress on Children’s Environmental Health: A Call to Action* is available for download from the site. Also accessible from the initiative homepage is an overview presentation with additional background information on the need for indicators in this area and on the information such indicators could yield. The overview presentation also looks at options that can be pursued in implementing pilot projects.

The Regional Initiatives link on the homepage takes visitors to an introduction to the seven regional pilot programs that have been set up. Many of these programs began in the past 18 months. This page describes how the pilot programs function and puts forth ideas on how they can best be managed. Clicking on a region name takes visitors to information on which agencies lead the programs, the status of the programs, and how indicators have been developed, if this has been accomplished. Related Links at the bottom of each pilot project page include lists of entities that have agreed to partner with the initiative. These include governmental agencies, international organizations such as the UN Environment Programme, NGOs, and others.

The global initiative will work to help meet the goals of another project launched at the summit, the Healthy Environments for Children Alliance. The mission of this alliance is to reduce environmental risks to children’s health through education, increasing political will, mobilizing resources, and fostering focused and urgent action on this issue.

## Figures and Tables

**Figure:**